# Expression and Functional Contribution of Different Organic Cation Transporters to the Cellular Uptake of Doxorubicin into Human Breast Cancer and Cardiac Tissue

**DOI:** 10.3390/ijms23010255

**Published:** 2021-12-27

**Authors:** Marcus Otter, Susanne Csader, Markus Keiser, Stefan Oswald

**Affiliations:** 1 Department of Pharmacology, University Medicine of Greifswald, 17489 Greifswald, Germany; marcus.otter@yahoo.de (M.O.); susanne.csader@uef.fi (S.C.); markus.keiser@web.de (M.K.); 2 Institute of Pharmacology and Toxicology, University Medical Center Rostock, 18051 Rostock, Germany

**Keywords:** doxorubicin, transporter, cardiotoxicity, breast cancer

## Abstract

Doxorubicin is a frequently used anticancer drug to treat many types of tumors, such as breast cancer or bronchial carcinoma. The clinical use of doxorubicin is limited by its poorly predictable cardiotoxicity, the reasons of which are so far not fully understood. The drug is a substrate of several efflux transporters such as P-gp or BCRP and was recently reported to be a substrate of cation uptake transporters. To evaluate the potential role of transporter proteins in the accumulation of doxorubicin at its site of action (e.g., mammary carcinoma cells) or adverse effects (e.g., heart muscle cells), we studied the expression of important uptake and efflux transporters in human breast cancer and cardiac tissue, and investigated the affinity of doxorubicin to the identified transporters. The cellular uptake studies on doxorubicin were performed with OATP1A2*1, OATP1A2*2, and OATP1A2*3-overexpressing HEK293 cells, as well as OCT1-, OCT2-, and OCT3- overexpressing MDCKII cells. To assess the contribution of transporters to the cytotoxic effect of doxorubicin, we determined the cell viability in the presence and absence of transporter inhibitors in different cell lines. Several transporters, including P-gp, BCRP, OCT1, OCT3, and OATP1A2 were expressed in human heart and/or breast cancer tissue. Doxorubicin could be identified as a substrate of OCT1, OCT2, OCT3, and OATP1A2. The cellular uptake into cells expressing genetic OATP1A2 variants was markedly reduced and correlated well with the increased cellular viability. Inhibition of OATP1A2 (naringin) and OCT transporters (1-methyl-4-phenylpyridinium) resulted in a significant decrease of doxorubicin-mediated cytotoxicity in cell lines expressing the respective transporters. Similarly, the excipient Cremophor EL significantly inhibited the OCT1-3- and OATP1A2-mediated cellular uptake and attenuated the cytotoxicity of doxorubicin. In conclusion, genetic and environmental-related variability in the expression and function of these transporters may contribute to the substantial variability seen in terms of doxorubicin efficacy and toxicity.

## 1. Introduction

The anthracycline antibiotic doxorubicin is an antineoplastic drug frequently used for the effective treatment of many solid tumors, including lung, ovarian, gastric, and breast cancer, as well as hematological malignancies such as lymphoma, acute leukemia, and multiple myeloma. The cytotoxic mode of action of doxorubicin is complex, and includes its intercalation into the DNA, leading to DNA strand breakage, inhibition of DNA- and RNA-polymerases, inhibition of DNA-topoisomerase II, and formation of free radicals. Despite its established use for decades and its considerable side effects, including bone marrow suppression (i.e., immunosuppression), nausea, vomiting, diarrhea, hemorrhagic cystitis, and alopecia, the drug remains an indispensable component of modern chemotherapy protocols [1,2,3,4].

One of the most serious and dose-limiting side effects of doxorubicin is irreversible cardiotoxicity leading to dilated cardiomyopathy and congestive heart failure, the reasons of which are still not fully understood [3,5,6,7]. Doxorubicin is assumed to cause cardiomyopathy by inducing oxidative stress (via the formation of free radicals) and p53-mediated apoptosis. To avoid doxorubicin-related cardiotoxicity, the cumulative dose may not exceed 450–600 mg/m^2^, which is still associated with a risk of congestive heart failure of about 3–18% [8,9]. However, lower doses of the drug may negatively affect doxorubicin efficacy. Unfortunately, the therapeutic benefit from current preventive strategies using dexrazoxane is rather limited and is also said to worsen myelosuppression and to weaken the antitumor activity of doxorubicin [5,6].

Considering that cardiotoxicity requires the accumulation of doxorubicin in cardiomyocytes, processes that mediate the cellular uptake into heart tissue are of great interest. The same is true for reaching adequate drug concentrations at the desired site of action, i.e., tumor cells. In this regard, it was hypothesized that enrichment of the anthracycline was achieved by passive diffusion [10]. However, since doxorubicin is a weak base which is almost completely present as cationic moiety under physiological conditions (i.e., 89% at pH 7.4) and has a low membrane permeability despite its fair solubility (i.e., BCS classification: III), transport proteins are expected to be involved in the transport of doxorubicin across biological membranes [11]. An additional argument for the involvement of transport proteins is the saturable cellular uptake as observed in in vitro studies [12]. In this regard, it is well established that doxorubicin is a substrate of the ATP-binding cassette (ABC) efflux transporters P-glycoprotein (P-gp), breast cancer resistance protein (BCRP), and multidrug resistance-associated proteins (MRPs), which were said to cause drug resistance in cancer therapy by reducing intracellular concentrations of doxorubicin [13,14,15,16,17,18,19,20]. 

On the contrary, the knowledge about cellular uptake transporters of the solute carrier family is much more limited. Here, the organic cation transporters (OCT) OCT3, OCT6, OCTN1, and the organic anion transporting polypeptide (OATP) 1A2 have been described to mediate the cellular uptake of doxorubicin [21,22,23,24,25,26,27]. Quite recently, chemical and genetic knockout of OCT3 was demonstrated to attenuate doxorubicin-related cardiac injury in vitro and in vivo [23]. 

Thus, there seems to be a complex interplay of cellular uptake and efflux transport of doxorubicin determining its tissue exposure at the site of desired action (tumor cells) and at the site of unwanted adverse effects (cardiomyocytes). Endogenous and environmental factors may affect uptake and efflux carrier expression and function, resulting in high inter-subject variability (e.g., genetic polymorphisms or drug-drug interactions). Consequently, expression data of uptake and efflux transporters in tissues involved in doxorubicin efficacy and safety, together with functional transport data and their relevance for the cytotoxic activity of doxorubicin, are required to estimate individual transporter contribution to the net cellular uptake of doxorubicin. In contrast to the previous studies, which focused in a very dedicated manner on the transport of doxorubicin by OATPs such as OATP1A2 [22] or OCT3 [23], our study aims to combine the available knowledge to deduce the relevance of transporter expression and function for the accumulation of doxorubicin at its site of action and its site of unwanted effects. Moreover, inter-subject variability caused by genetic polymorphisms and drug-drug interactions will be considered. 

In order to get deeper insights into the functional relevance of different transporters for the accumulation of doxorubicin in different tissues, we (1) characterized the expression of reported doxorubicin transporters in human breast cancer and heart tissue as well as in established breast cancer cell lines, (2) studied the cellular uptake of doxorubicin by different cation transporters (i.e., OCT1-3, OATP1A2), (3) determined the impact of genetic polymorphisms of *SLCO1A2* (encoding for OATP1A2) on the cellular uptake of doxorubicin, and (4) investigated the cytotoxic effects of doxorubicin independent of genetic polymorphisms of *SLCO1A2* and inhibition of OATP1A2 and OCT transporters.

## 2. Results

### 2.1. Gene Expression of Transporters

**Figure 1:**
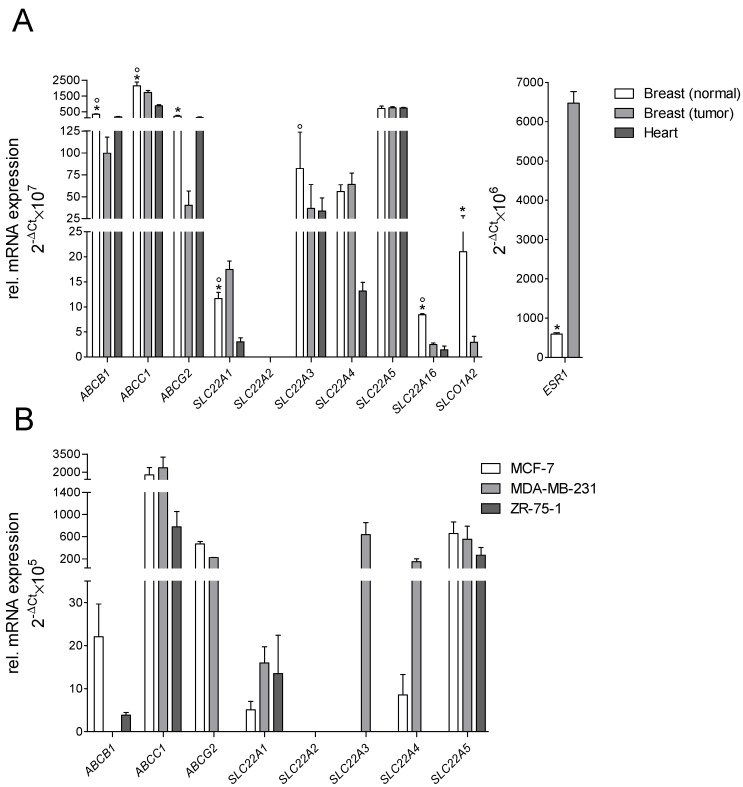
Relative mRNA expression of uptake and efflux transporters: (**A**) in human heart, normal and tumorous breast tissue; 5-donor-pooled sample; (**B**) in breast cancer cell lines MCF-7, MDA-MB-231, and ZR-75-1 (each of 3 measurements in duplicate, reference genes: 18S/GAPDH; * for significant differences between normal and tumorous breast tissue, ° for significant differences between normal breast and cardiac tissue).

On the mRNA level, the efflux transporters *ABCB1*, *ABCC1*, and *ABCG2* were found expressed in human heart, in normal and tumorous breast tissue, and in human breast cancer cell lines MCF-7, MDA-MB-231, and ZR-75-1 (Figure 1). Of the solute carrier family, *SLC22A1*, *SLC22A3*, *SLC22A4*, *SLC22A5*, and *SLC22A16* were expressed in all tissues. *SLCO1A2* was not expressed in the human heart but in breast tissue, while *SLC22A2* was not detectable in any tissue. Expression of *ABCB1*, *ABCC2*, *ABCG2*, *SLC22A16*, and *SLCO1A2* was significantly higher in normal breast tissue compared to tumorous tissue, while *SLC22A1* and the tumor marker *ESR1* showed a significantly higher expression in breast cancer tissue. In breast cancer cell lines, *SLC22A1* was only expressed in MCF-7 and ZR-75-1 cells, while SLC22A3 was exclusively expressed in MDA-MB-231 cells. *SLC22A2*, *SLC22A16*, and *SLCO1A2* were not expressed in any of the investigated breast cancer cell lines (Ct values > 35).

### 2.2. Cellular Transport of Doxorubicin

**Figure 2:**
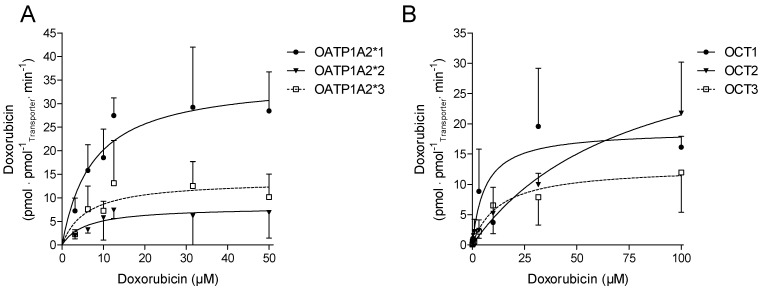
(**A**) Uptake of doxorubicin into stably transfected HEK293 cells expressing OATP1A2*1, *2, *3, and (**B**) into MDCKII cells stably expressing OCT1, 2 and 3 (data given as mean ± SD; *n* = 3).

Doxorubicin showed no cytotoxic effects, within one hour, on transfected cell lines or on tumorous cell lines at the concentrations tested (data not shown). In stably transfected cells expressing OATP1A2, OCT1, OCT2, and OCT3, doxorubicin was taken up in a time-dependent manner within the incubation time of up to 30 min. The concentration-dependent OATP1A2-, OCT1, and -3 mediated uptake of doxorubicin, was saturated within the incubation time of 5 and 3 min, respectively, while the OCT2-mediated uptake was not saturated (Figure 2). The resulting Michaelis-Menten constants (Km) and the maximal uptake rate (Vmax) values for the cellular uptake of doxorubicin are summarized in Table 1. 

Among the OCTs investigated in this study, doxorubicin showed the highest affinity for OCT1, followed by OCT3 and OCT2. The highest capacity (Vmax) for doxorubicin transport was observed for OCT2, while OCT1 and OCT3 showed markedly lower capacities, respectively (Table 1). OATP1A2 was shown to be a high-affinity uptake transporter for doxorubicin. The affinity of doxorubicin to OATP1A2 was lower than for OCT1, but higher than for OCT2 and OCT3. The capacity of OATP1A2 was comparable to that of OCT2. With respect to the intrinsic clearance, the rank order of the investigated in vitro transport of doxorubicin was OATP1A2 > OCT1 > OCT3 and OCT2 (Table 2). The genetic variants OATP1A2*2 and *3 showed a considerably lower cellular uptake of doxorubicin than the wild type of OATP1A2*1 (Figure 2A and Table 1).

In Transwell^®^ assays using stably transfected cells expressing P-gp or BCRP, doxorubicin showed a vectorial transport from the basolateral to the apical compartment, which was markedly reduced in the presence of the P-gp inhibitor PSC833 or the BCRP inhibitor Ko143, respectively (Supplemental Figure S1).

### 2.3. Cell Viability

**Figure 3:**
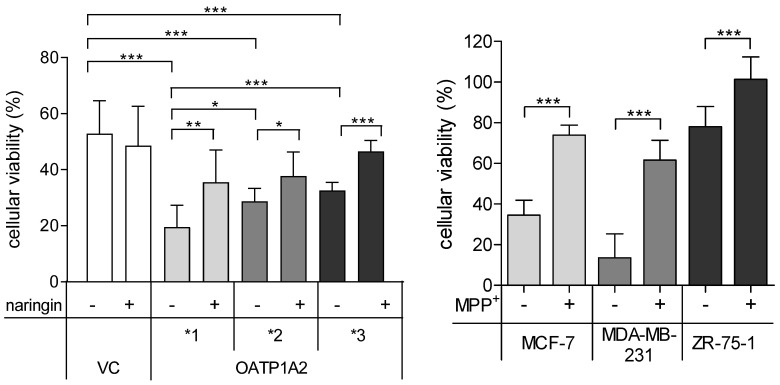
Cell viability of vector transfected (VC) HEK293 cells or cells expressing OATP1A2*1, *2, *3 after incubation with 100 µmol/L doxorubicin for 72 h in presence (+) or absence (−) of 500 µM naringin (left panel). Cell viability of breast cancer cell lines after incubation with 100 µmol/L doxorubicin for 72 h in presence (+) or absence (−) of 100 µM 1-methyl-4-phenylpyridinium (MPP+) (right panel). Viability was compared to cells incubated with DMSO as solvent for doxorubicin (=100%) (data given as mean ± SD; *n* = 3; * *p* < 0.05; ** *p* < 0.01; *** *p* < 0.001).

In the presence of doxorubicin, cell viability was significantly reduced in OATP1A2 transfected cells and breast cancer cell lines (Figure 3). In OATP1A2*2 and *3 transfected cells, doxorubicin-mediated cytotoxicity was significantly lower than in MDCKII-OATP1A2*1 cells. The cytotoxic effects of doxorubicin were significantly attenuated by naringin in OATP1A2 transfected cells and by MPP+ in breast cancer cell lines (Figure 3). 

**Figure 4:**
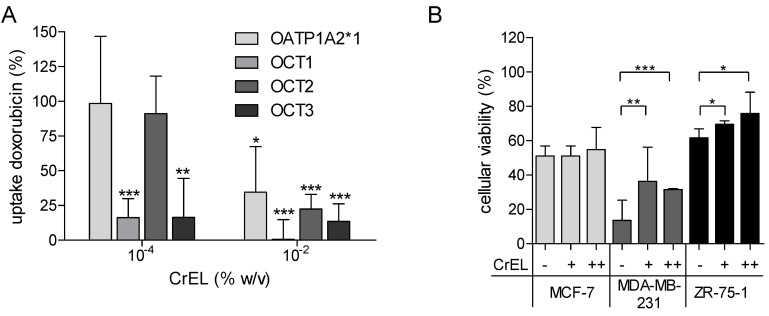
(**A**) Competition of doxorubicin and CrEL in HEK293-OATP1A2 and MDCKII-OCT1, -OCT2, -and OCT3 cells after incubation with doxorubicin (10 µM for OATP1A2, 50 µM for OCTs) in presence (+) or absence (−) of CrEL and an incubation time of 2 min (OCTs) or 3 min (OATP1A2). (**B**) Cell viability assay in in breast cancer cell lines after incubation with 100 µM doxorubicin for 72 h in presence (+/++) or absence (−) of CrEL (+, 10^−4^ %; ++, 10^−2^ %). Viability was compared to cells incubated with DMSO as solvent for doxorubicin (=100%) (data gives as mean ± SD; *n* = 3; * *p* < 0.05; ** *p* < 0.01; *** *p* < 0.001).

The doxorubicin uptake was significantly reduced to 0.57–35% in the presence of 0.01% CrEL in cells stably expressing OATP1A2 and OCT1-3 (Figure 4A). In breast cancer cell lines incubated with doxorubicin, increasing concentration of CrEL showed a protective effect against cytotoxicity in MDA-MB-231 and ZR-75-1 cells (Figure 4B).

## 3. Discussion

The clinical use of doxorubicin is limited by its high variability in terms of efficacy and safety [3,4,28]. Concerning the latter, dose-dependent cardiotoxicity remains a challenge associated with doxorubicin-containing treatment protocols [5,6,7]. There is convincing evidence that the cellular accumulation of poorly permeable and cationic doxorubicin at the site of action (tumor cells) and of side effects (cardiomyocytes) is determined by drug transporting proteins [11,12]. In this regard, ABC transporters such as P-gp and BCRP are well known to be involved in the pharmacokinetics of doxorubicin and may affect its therapeutic efficacy [13,14,15,18,19,20]. However, therapeutic approaches to improve the outcome of cancer patients by co-administering inhibitors of P-gp were not successful, suggesting that alternative mechanisms may be involved in the variability of tissue exposure and drug resistance, such as compromised transporter-mediated drug uptake [29,30]. Recently, more attention has been paid to the cellular uptake processes of doxorubicin by SLC transporters [16]. Accordingly, the cation transporters OCT1, OCT3, OCT6, OCTN1, and OATP1A2 were shown to be involved in the in vitro or in vivo transport of doxorubicin [21,22,23,24,25,26,27,31]. 

Associated with this, our expression analysis demonstrated that in addition to the efflux transporters P-gp (*ABCB1*), MRP1 (*ABCC1*), and BCRP (*ABCG2*), the uptake carriers OCT1 (*SLC22A1*), OCT3 (*SLC22A3*), OCTN1 (*SLC22A4*), OCTN2 (*SLC22A5*) and OCT6 (*SLC22A16*) were also expressed in human breast and cardiac tissue, which is in line with previous findings [19,23,32,33,34,35,36,37,38]. OATP1A2 (*SLCO1A2*) could only be detected in human breast but not cardiac tissue, which agrees with the literature [39,40,41]. Our data also agree with public data available in the Human Protein Atlas (http://www.proteinatlas.org; accessed on 15 October 2021). 

Interestingly, except for OCT1, all investigated transporters were equally (OCTN1-2) or even significantly lower expressed in breast cancer tissue (i.e., P-gp, MRP1, BCRP, OCT3, OCT6, and OATP1A2) compared to the tissue of healthy controls. To confirm the integrity of our analysis, the breast cancer marker gene estrogen receptor 1 (ESR1) was analyzed in parallel and confirmed markedly higher expression levels in the pooled breast cancer tissue as expected. These findings contrast some former studies in which cancer was reported to cause substantial up-regulation of transporter protein expression [19,39,42]. However, other studies could not verify this aspect of tumor-related transporter induction [36,43,44,45]. This inconsistency might be caused by the different origin of tumor cells (e.g., primary tumor or metastasis), anticancer drugs used that may affect transporter regulation, inflammatory processes, or epigenetic mechanisms (i.e., miRNA, DNA methylation), which were shown to contribute considerably to transporter regulation in cancer cells [46]. 

Except for OCT6 and OATP1A2, all transporters observed in human breast cancer tissue could also be detected in established human breast cancer cell lines. In contrast to this, Okabe et al. detected OCT6 in MCF-7 cells, but at low expression levels [26]. Another study supports our findings on the absence of OATP1A2 in the investigated breast cancer cells [45]. As a noteworthy limitation of our expression analysis, we generated only gene expression data (due to the small tissue amount), which did not necessarily correlate to the encoded proteins.

The identified transporter might be involved in the doxorubicin accumulation in human heart and breast cancer tissue. Indeed, our study demonstrated that OCT1-3 and OATP1A2 are cellular uptake transporters of doxorubicin with Km values ranging from 5–13 µmol/L. Considering that plasma concentrations of doxorubicin during chemotherapy of up to 600 ng/mL (~1 µmol/L) are reached, these transporters may also be relevant in vivo. 

The observation that doxorubicin is a substrate of OCT1-3 and OATP1A2 confirms previously published data [21,22,23,31]. Our observed transport kinetics for OATP1A2 differ somewhat from the data from Lee et al., who reported a Km value of 31.7 µmol/L. These differences may be due to the use of different cellular in vitro systems (transiently transfected HeLa cells vs. stably transfected HEK293 cells) [22]. 

It is established that OATP1A2 is a genetically polymorphic protein resulting in functionally relevant changes in the respective transport function [47,48]. We therefore included the frequently occurring polymorphisms SLCO1A2*2 (38T > C, rs10841795) and SLCO1A2*3 (516A > C, rs11568563) with allele frequencies of 2–16% to our analysis. We observed markedly diminished cellular uptake clearance (51–70%) by the genetic variants, which is in line with a previous study [22]. In parallel to the decreased cellular uptake of doxorubicin into OATP1A2*2/*3 cells, the cytotoxic effect of doxorubicin was also significantly attenuated compared to that in OATP1A2*1 cells. This suggests that genetic variability may contribute to reduced cellular uptake and the efficacy of doxorubicin in malignant target tissues, as shown for other substrates of OATP1A2 such as imatinib or methotrexate [49]. On the contrary, OATP1A2 is not expected to contribute to doxorubicin accumulation in cardiac tissue, and thus cardiotoxicity, because it is not expressed in cardiomyocytes as shown by our study and others [23]. In line with this assumption, Durmus et al. demonstrated similar cardiac tissue levels of doxorubicin in Oatp1a/1b^−/−^ and wild-type mice despite higher plasma concentrations in Oatp1a/1b knockout mice [21].

The observed cellular uptake clearances (OATP1A2 > OCT1 > OCT3 > OCT2) along with gene expression data in breast cancer (OCT3 > OCT1 > OATP1A2; OCT2 not expressed) and cardiac tissue (OCT3 > OCT1; OCT2 and OATP1A2 not expressed) point to an outstanding role of OCT3 in both efficacy and safety. However, distinct protein abundance data are needed to conclude the clinical relevance using in vitro- in vivo extrapolation approaches [50]. At any rate, our hypothesis is in line with a very recent study from Huang et al., which demonstrated in vitro using stem-cell-derived cardiomyocytes and in vivo (mouse model) that OCT3 is expressed in cardiomyocytes and mediates accumulation of doxorubicin in heart tissue [23]. Consequently, genetic knockout and chemical inhibition of OCT3 resulted in attenuated doxorubicin-induced cardiac injury. In addition, OCTN1 may also be of clinical importance for mediating cardiotoxicity of doxorubicin as this transporter is even more highly expressed in human cardiomyocytes than OCT3 and seems to be also a potent uptake transporter [23,26]. Unfortunately, the lack of distinct affinity data does not allow further estimations. Moreover, OCT1 and OCT6 may also contribute to cardiac accumulation of doxorubicin and the respective organ toxicity considering the considerable expression of both carriers in human heart tissue and their comparably high affinity to doxorubicin (Km ~5 µM) [23,24,51].

On the other side, OCT2 does not play any role in the cellular uptake of doxorubicin in human heart or breast cancer tissue because the transporter is absent in both organs. It can be speculated that OCT2 may be involved in the renal excretion of the drug, while the hepatic uptake is mediated by OCT1. 

Taking into account that the aforementioned cation transporters are involved in the cellular uptake of doxorubicin and are expressed in several tumor entities, the following scenarios of variability in drug efficacy and safety may occur: (1) genetic variability in the expression and function as discussed for OATP1A2; (2) increased or decreased cellular uptake of doxorubicin due to disease- or drug-related up- or down-regulation of the respective transporter(s) (very little data available) [39,52]; and (3) reduced cellular uptake caused by transporter inhibition, namely unwanted drug-drug interactions.

While the latter aspect may benefit the prevention of serious side effects such as cardiotoxicity [16], decreased doxorubicin concentration in tumor cells may threaten the therapeutic drug effects.

In this regard, our study demonstrated that the OATP1A2 inhibitor naringin and the unspecific OCT inhibitor MPP reduced the cellular uptake, and in turn, the cytotoxicity of doxorubicin in OATP1A2-overexpressing cells and breast cancer cell lines, respectively. Considering the numerous inhibitors of OCT transporters and OATP1A2 [53,54], their combined use with doxorubicin in tumor patients appears to be realistic, and may result in decreased susceptibility of tumor cells to doxorubicin. However, clinical data that confirm this hypothesis are not yet available. 

A clinically possible scenario for such an unwanted drug-drug interaction might be the combination with Cremophor EL (CrEL), which is a solubilizing agent and a known modulator of P-gp and CYP3A4 [55]. This pharmaceutical excipient is used in relatively high doses to enhance the solubility of the poorly water-soluble cytostatic drug paclitaxel (e.g., Taxol^®^, paclitaxel formulation with 527 mg/mL CrEL), which is frequently combined with doxorubicin in breast cancer patients [56]. 

Our inhibitory in vitro studies demonstrated the significant and dose-dependent reduction of the cellular doxorubicin uptake in OATP1A2- and OCT1-3-transfected cells in the presence of CrEL at concentrations reached in vivo using recommended doses of paclitaxel [55,57]. These data suggest that CrEL may compromise the cytotoxic effects of doxorubicin. Indeed, our additional accumulation studies of breast cancer cells showed increased viability, i.e., decreased cytotoxicity of doxorubicin in MDA-MB-231 and ZR-75-1 cells, but not in MCF-7 cells, which was also reported by Gianni et al. [58]. The reason for these differences between the cell lines may be explained by the lack of OCT3 and the low expression of OCT1 in MCF-7 cells. One may also speculate about the potential contribution of P-gp to the cellular accumulation of doxorubicin because doxorubicin is a substrate of P-gp, which can be inhibited by CrEL [55]. Thus, P-gp inhibition would be expected to raise intracellular concentrations of doxorubicin. On the contrary, treatment of MCF-7 TH cells with CrEL even decreased cellular concentrations of the drug, most likely through the inhibition of uptake transporters [58]. 

However, P-gp seems to play a relevant role concerning the level of global doxorubicin pharmacokinetics, as indicated by the strikingly increased serum concentrations of doxorubicin in combination with CrEL and paclitaxel, most likely explained by the inhibition of biliary and renal P-gp [58]. Interestingly, this interaction could only be observed when paclitaxel and CrEL were administered before doxorubicin.

According to the product information of doxorubicin, the risk for cardiotoxicity was also found to be increased if there is no temporal distance of at least one hour between the administration of doxorubicin and subsequent paclitaxel, which does not support the hypothesis that inhibition of OCT transporters may prevent cardiac injury. 

However, it remains very difficult to distinguish between potentially lowered uptake of doxorubicin into cardiac tissue due to CrEL and the additional cardiotoxicity as caused by paclitaxel [59]. The same is true for the cytostatic effects, because despite potential inhibition of OCT 1/3 and OATP1A2-mediate uptake of doxorubicin into tumor cells by CrEL, paclitaxel adds synergistically to the anticancer activity.

In conclusion, our study confirmed the previously shown involvement of OCT1-3 and OATP1A2 as well as P-gp and BCRP in the complex net uptake of doxorubicin into cells involved in its desired pharmacological action (e.g., breast cancer cells) and toxicity (e.g., cardiomyocytes). Individual data about the distinct protein abundance of transporters involved in the pharmacokinetics and tissue distribution (P-gp, BCRP, MRPs, OCT1, OCT3, OCT6, OCTN1, and OATP1A2) are needed in addition to pharmacogenetic information to conclude on their impact on the efficacy and safety of the drug. Considering the complexity of doxorubicin transport and the potential individual variability in transporter expression and function, it remains very challenging to predict distinct tissue levels of the drug even when using highly sophisticated modeling and prediction approaches.

## 4. Materials and Methods

*Chemicals:* Doxorubicin (Adrimedac^®^, injectable solution, 2 mg/mL) was purchased from Pharmachemie B.V. (Haarlem, Netherlands). PSC833 was kindly provided by Novartis (Nuremberg, Germany). Ko143, sodium dodecyl sulfate (SDS), and ethylenediaminetetraacetic acid (EDTA) were obtained from Sigma-Aldrich (Taufkirchen, Germany).

*Gene expression analysis:* Total RNA from normal heart, normal breast, and tumorous breast tissue from a 5 donor pool were purchased from Biochain (Newark, CA, USA). mRNA was isolated from all three breast cancer cell lines using the NucleoSpin RNA II kit (Macherey-Nagel, Düren, Germany). The quantity and purity of RNA were analyzed by a NanoDrop ND-1000 spectrophotometer (NanoDrop Technologies, Wilmington, DE, USA). The RNA integrity was proved via the Agilent 2100 Bioanalyzer (Agilent Technologies, Santa Clara, CA, USA). cDNA was synthesized from total and from mRNA using the High Capacity RNA-to-cDNA kit (Life Technologies, Darmstadt, Germany) according to the manufacturer’s instructions. Gene expression levels of uptake and efflux transporters and of endogenous control genes were investigated using a TaqMan^®^ Custom Array (Table 2) on a 7900HT Sequence Detection System (Life TechnologiesTM, Darmstadt, Germany). The most suitable reference genes or combinations of reference genes for gene expression analysis were determined using the BestKeeper^®^ algorithm [60].

*Cell culture:* All cell lines were grown at 37 °C, 95% humidity, and 5% CO_2_ unless otherwise noted. Media and supplements were purchased from PAN-Biotech (Aidenbach, Germany). Human embryonic kidney 293 (HEK293) and Madin Darby canine kidney (MDCK) II cells were obtained from the European Collection of Cell Cultures (Salisbury, United Kingdom). HEK293 cells were grown in a minimal essential medium supplemented with 10% fetal bovine serum, 2 mM L-glutamine, and 2 mM nonessential amino acids. MDCKII cells were grown in Dulbecco’s modified Eagle’s medium supplemented with 10% fetal bovine serum and 4 mM L-glutamine. Cells were grown until reaching a confluence of 90%. HEK293 cells stably transfected with human OATP1A2*1, *2, *3, or MDCKII cells stably expressing OCT1, OCT2, OCT3, P-gp, and BCRP, and the respective wild-type control cells were established as previously described [61,62,63]. Human breast cancer cell lines MDA-MB-231, MCF-7, and ZR-75-1 were obtained from the American Type Culture Collection (Manassas, VA, USA). MCF-7 cells were grown in minimal essential medium supplemented with 10% fetal bovine serum and 0.01 mg/mL human insulin. ZR-75-1 cells were grown in RPMI-1640 medium supplemented with 10% fetal bovine serum. MDA-MB-231 cells were grown in Leibovitz’s L-15 Medium supplemented with 10% fetal bovine serum at 37 °C, and 95% humidity.

*Cellular uptake studies:* For all transport studies, cells were seeded in 24-well plates and incubated in a full growth medium at an initial density of 200,000 cells/well for 3 days. Before starting each transport experiment, cells were washed with a pre-warmed (37 °C) incubation buffer [64]. After incubation with doxorubicin, cells were washed three times with an ice-cold incubation buffer and lysed with 0.2% SDS and 5 mM EDTA. Protein concentration of whole cell lysate was determined to quantify cell density after each experiment using the BCA assay according to the manufacturer’s instructions (Pierce, Rockford, IL, USA).

Time-dependent doxorubicin uptake into OATP1A2*1 and OCT1-3 transfected cells was measured using 100 µmol/L doxorubicin over an incubation period of 0.5 to 30 min. The Michaelis–Menten constant (Km) and the maximal uptake rate (Vmax) values for the cellular uptake of doxorubicin (0–100 µmol/L) were determined after an incubation time of 5 min for OATP1A2*1 transfected cells and 3 min for cells stably expressing OCT1-3. Doxorubicin was quantified in cell lysates using LC-MS/MS. Protein abundance of OATP1A2*1–*3 and OCT1–3 were measured by mass spectrometry-based targeted proteomics using a validated LC-MS/MS method as recently described [65]. In competition assays, OATP1A2 transfected cell lines, as well as breast cancer cell lines, were incubated with 100 µmol/L doxorubicin in the presence or absence of naringin (500 µmol/L), 1-methyl-4-phenylpyridinium (MPP+, 100 µmol/L) or Cremophor EL (CrEL, 0.0001–0.01%). The lack of cytotoxicity of doxorubicin (100 µmol/L) within the 30 min incubation time was confirmed using the Presto Blue™ cell viability assay (Life Technologies, Darmstadt, Germany). 

*Quantitative assay for doxorubicin:* Doxorubicin was measured by a validated liquid chromatography-tandem mass spectrometry (LC-MS/MS) method in the range of 0–1000 ng/mL. 200 μL of each cell lysate sample was mixed with 25 μL internal standard (5 µg/mL daunorubicin), 25 μL citric acid (1%), and 400 μL ice-cold acetonitrile for protein precipitation. After intensive mixing, samples were subjected to vacuum centrifugation for 10 min at 11,400× *g* and room temperature. The clear supernatant was transferred to HPLC vials. The measurement and quantitative evaluation were carried out using the LC-MS/MS system QTRAP 4000^®^ (AB SCIEX, Darmstadt, Germany) and the Software Analyst^®^ 1.4 (Applied Biosystems, Darmstadt, Germany).

*Resazurin-Assay:* To determine cell viability, cells were seeded at a density of 10,000 cells/well in 96-well multiplates. On day three, cells were incubated with doxorubicin (100 µmol/L) in the presence or absence of naringin (500 µmol/L), MPP+ (100 µmol/L) or CrEL (0.0001–0.01%). After 72 h, a doxorubicin-containing medium was removed, replaced by fresh medium and resazurin (PromoCell, Heidelberg, Germany) that was added to a final concentration of 10% followed by incubation at 37 °C for three hours. Afterward, 100 µL from each well was placed in a cavity of a 96-well plate, and the fluorescence was determined on a microplate reader (TECAN infinite M200, Tecan GmbH, Crailsheim, Germany) at an excitation wavelength of 530 nm and an emission wavelength of 590 nm. Data were calculated as the percentage of the cell viability of DMSO (solvent for doxorubicin) treated cells.

*Transwell-Assays:* P-gp-and BCRP-mediated transport of doxorubicin was measured using the Transwell^®^ technique. In brief, MDCKII cells stably expressing P-gp or BCRP were seeded on 0.4 µm Transwell^®^ membrane inserts (200.000 cells/insert; Ø6.5 mm, growth area 0.33 cm_2_) and allowed to differentiate for five days. The integrity of the monolayer was confirmed by monitoring the trans-epithelial electrical resistance (TEER) using the EVOMX system (WPI, Sarasota, FL, USA). Before each experiment, cells were washed with a pre-warmed (37 °C) incubation buffer and incubated for 30 min at 37 °C. Afterward, 0.75 or 0.25 mL of a 100 μmol/L doxorubicin solution, respectively, were added to the basal or apical donor compartment in the presence or absence of the P-gp inhibitor PSC833 (10 µmol/L) or the BCRP inhibitor Ko143 (1 µmol/L). Every 30 min, the incubation buffer was removed from the receiver compartment and replaced by a freshly preheated buffer for a total time of 2.5 h. The concentrations of doxorubicin in the receiver compartment were measured using the LC-MS/MS method described above.

Statistical evaluation: All experiments were performed at least three times unless otherwise noted. The OATP1A2*1–*3 and OCT1-3 net uptake was obtained by subtracting the uptake into wild-type cells from that of the transporter-transfected cells. Km and Vmax were assessed using Prism 5.01 (GraphPad Software, San Diego, CA, USA). The differences in the doxorubcin uptake in OATP1A2*1–*3 and in OCT1-3 transfected cells were adjusted to the membranous transporter protein levels in the whole cell lysate. Km and Vmax are presented as arithmetic means ± standard deviation. Statistical significance in cell viability assays was established with the t-test. Apparent permeability (Papp) and the efflux ratio (ER) were calculated as mentioned before [66].

## Figures and Tables

**Table 1:** Kinetic data for the doxorubicin uptake in stable transfected HEK293 cells expressing OATP1A2*1, *2 or *3 and MDCKII cells expressing OCT1, OCT2, or OCT3.

Transporter	Km (µmol/L)	Vmax (pmol/mg × min)	Clint (µL/mg × min)
OATP1A2*1	7.49 ± 3.41	35.3 ± 5.23	4.71 ± 1.53
OATP1A2*2	5.84 ± 3.95	8.09 ± 2.55	1.39 ± 0.65
OATP1A2*3	5.94 ± 3.07	13.8 ± 3.40	2.32 ± 0.99
OCT1	4.66 ± 3.43	19.4 ± 3.78	4.16 ± 1.10
OCT2	76.1 ± 42.8	37.8 ± 11.1	0.50 ± 0.26
OCT3	13.0 ± 10.7	12.9 ± 3.60	0.99 ± 0.33

**Table 2:** Gene assays used for real-time PCR.

Gen	Protein	Assay-ID
*GAPDH*	GAPDH	Hs99999905_m1
*18S*	18S	Hs99999901_s1
*ESR1*	ERα	Hs01046816_m1
*ABCB1*	P-gp	Hs01067802_m1
*ABCC1*	MRP1	HS01561502_m1
*ABCG2*	BCRP	Hs01053790_m1
*SLC22A1*	OCT1	Hs00427552_m1
*SLC22A2*	OCT2	Hs01010723_m1
*SLC22A3*	OCT3	Hs01009568_m1
*SLC22A4*	OCTN1	HS01548718_m1
*SLC22A5*	OCTN2	HS00929869_m1
*SLC22A16*	OCT6	HS00263925_m1
*SLCO1A2*	OAPT1A2	Hs00366488_m1

## Supplementary Materials

The following are available online at https://www.mdpi.com/article/10.3390/ijms23010255/s1.
