# Supplementary material for: Expression and Functional Contribution of Different Organic Cation Transporters to the Cellular Uptake of Doxorubicin into Human Breast Cancer and Cardiac Tissue
Source: Int J Mol Sci. 2021 Dec 27;23(1):255. doi: 10.3390/ijms23010255 (PMC8745601; doi:10.3390/ijms23010255)
Supplement: Supplementary file 1 [file ijms-23-00255-s001.zip › ijms-1477205-supplementary.pdf]

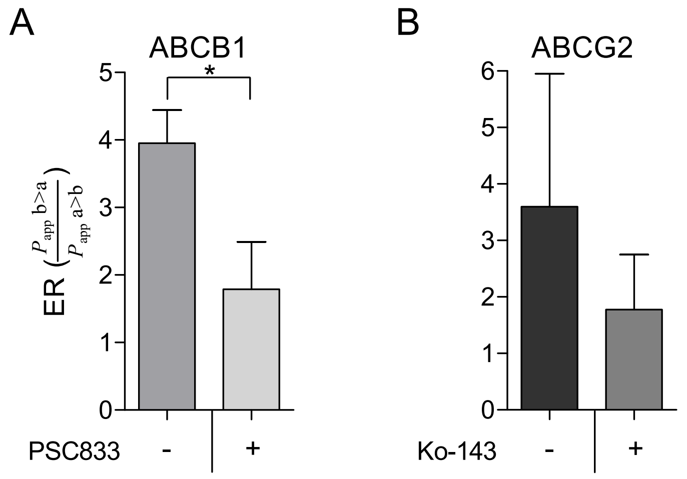

Figure S1: Calculated efflux ratios (ER) of the vectorial transport of doxorubicin in presence (+) or absence (-) of the ABCB1-inhibitor PSC833 or the ABCG2-inhibitor Ko143, respectively, in MDCKII cells stably transfected with **(A)** ABCB1 or **(B)** ABCG2. Cells were seeded on Transwell® filters and Papp-values were calculated for the basolateral (b) to apical (a) (P_{app} b>a) transport and vice versa (P_{app} a>b) of 100 µmol/L doxorubicin (data given as mean ± SD; n=3; *p<0.05).
